# Work participation, mobility and foot symptoms in people with systemic lupus erythematosus: findings of a UK national survey

**DOI:** 10.1186/s13047-019-0335-0

**Published:** 2019-04-29

**Authors:** M. J. Stevens, K. Walker-Bone, D. J. Culliford, B. Alcacer-Pitarch, A. Blake, N. Hopkinson, L. S. Teh, E. M. Vital, C. J. Edwards, A. E. Williams, L. Cherry

**Affiliations:** 1 MRC Lifecourse Epidemiology Unit, University of Southampton, Southampton, UK; 2 Arthritis Research UK/MRC Centre for Musculoskeletal Health and Work, Southampton, UK; 3 Methodological Hub, NIHR Collaboration for Applied Health Research and Care Wessex, University of Southampton, Southampton, UK; 4 NIHR Leeds Biomedical Research Unit, Leeds Teaching Hospitals NHS Trust and Leeds Institute of Rheumatic and Musculoskeletal Medicine, University of Leeds, Leeds, UK; 5 Private podiatrist, Verwood, Dorset UK; 6 Department of Rheumatology, Royal Bournemouth and Christchurch Hospitals NHS Foundation Trust, Bournemouth, UK; 7 Department of Rheumatology, Royal Blackburn Teaching Hospital, East Lancashire Hospital NHS Trust, Blackburn, UK; 8 Faculty of Clinical and Biomedical Sciences, University of Central Lancashire, Preston, UK; 9 NIHR Wellcome Trust Clinical Research Facility, University Hospital Southampton NHS Foundation Trust, Southampton, UK; 10 Directorate of prosthetics, orthotics and Podiatry, University of Salford, Salford, UK; 11 School of Health Sciences, Faculty of Health Sciences, University of Southampton, Building 45, Burgess Road, Southampton, SO17 1BJ UK; 12 Department of Podiatry, Solent NHS Trust, Southampton, UK

**Keywords:** Systemic lupus erythematosus, Foot, Mobility, Work, Employment, Survey

## Abstract

**Objective:**

The aim of this study was to investigate whether foot and lower limb related symptoms were associated with work participation and poor mobility in people with Systemic Lupus Erythematosus (SLE).

**Method:**

A quantitative, cross-sectional, self-reported survey design was utilised. People with SLE from six United Kingdom (UK) treatment centres and a national register were invited to complete a survey about lower limb and foot health, work participation and mobility. Data collected included work status and the prevalence of foot symptoms. The focus of the analyses was to explore potential associations between poor foot health work non-participation.

**Results:**

In total, 182 useable surveys were returned. Seventy-nine respondents reported themselves as employed and 32 reported work non-participation. The remaining were retired due to age or reported work non-participation for other reasons. Work non-participation due to foot symptoms was significantly associated with difficulty walking (*p* = 0.024), past episodes of foot swelling (*p* = 0.041), and past episodes of foot ulceration (*p* = 0.018). There was a significant increase in foot disability scores amongst those not working (mean 18.13, 95% CI: 14.85–21.41) compared to those employed (mean 10.16, 95% CI: 8.11–12.21).

**Conclusions:**

Twenty-nine% of people with SLE reported work non-participation because of lower limb or foot problems. Our results suggest that foot health and mobility may be important contributors to a persons’ ability to remain in work and should be considered as part of a clinical assessment.

## Introduction

Employment is a key measure of self-worth, as it grants independence and social esteem**.** Non-participation in work is associated with poorer health, higher rates of consultation in primary care and higher rates of indebtedness and mortality [1]. Musculoskeletal disorders are one of the two biggest causes of long-term work absenteeism in the developed world [2]. Inflammatory rheumatic disorders in particular have been shown to be associated with high levels of work disability such that 20–70% of people with rheumatoid arthritis have become work disabled within 5–10 years of symptom onset [3].

Baker and Pope systematically reviewed the published studies of work disability specifically in people with systemic lupus erythematosus (SLE) in 2009 [4] reporting rates of work non-participation in 32.5%. However, the reviewers commented on a heterogeneous literature, measuring work non-participation in a wide range of different ways, and highlighted a need for more high-quality research in this area in order to adequately determine the prevalence and causes of work non-participation in people with SLE.

A recent study by Cherry *et al* [5] has demonstrated that lower limb and foot problems are highly prevalent among people with SLE. Previous work has also shown that these symptoms are associated with substantial morbidity and functional impairment [5–9]; sixt-one% of people reported that foot pain adversely affected their lives [5]. However, to our knowledge, no research to date has specifically investigated the prevalence of, and relationship between, lower limb or foot related complications, poor mobility and work non-participation in people with SLE.

Therefore, this study seeks to explore the prevalence of self-reported work non-participation through SLE and foot symptoms in a sample of people living with SLE. The main aim and focus of the analyses was to explore potential associations between poor foot health and work non-participation.

## Methods

### Ethics, consent and permissions

The University of Salford (HSCR14/25) National Research Ethics Committee (14/SC/1009) granted ethical approval for the study. All research was completed in accordance with the declaration of Helsinki guidelines for research practice. Information about the purpose and intended use of the survey was included in a covering letter as part of the survey data sheet. Consent to take part in the survey was considered implicit following the completion and return of the form. Consent to publish was included.

### Study design

A quantitative, cross-sectional, self-reported survey design was utilised. This work was part of a larger national survey study, the methods and findings of which have been reported previously [10]. The current study aim was pre-specified as an intended sub-analysis of this larger survey. Data were collected regarding lower limb and foot health, foot pain, mobility and working status.

### Participants

Participants were included if they had a consultant confirmed diagnosis of SLE. The survey was distributed to all eligible people with SLE attending six UK NHS Rheumatology departments, where potential participants were being reviewed in dedicated specialist clinics (Blackburn, Christchurch, Leeds, Manchester, Salford and Southampton) and to members of the Lupus UK membership register via their newsletter.

### Outcome measures

The survey also included the Manchester Foot Pain and Disability Index (MFPDI) [11]. The MFPDI starts with the stem ‘Because of pain in my feet...’ followed by nineteen statements. Responders can select from three answers; ‘none of the time’ coded 0, ‘on some days’ coded 1, and ‘on most/every day’ coded 2. The last two items are tautological to the purposes of this study as they enquire about the impact of foot pain on work and leisure activities and were excluded from the current analyses. As suggested by Garrow and colleagues [11], the remaining 17 statements were summated into a simple score to give a total between 0 and 34, with higher scores indicative of more severe limitation. Moreover, we analysed the data using the three constructs of the MFPDI: mobility; scored between 0 and 20; pain; scored between 0 and 10 and personal appearance; scored between 0 and 4.

Our principal outcome measure was work non-participation (people who reported that they were currently on long-term sick leave or retired because of SLE or foot symptoms). Our reference population were respondents who reported that they were in either paid or voluntary employment.

### Analysis

Initial analyses were descriptive using IBM SPSS Statistics for Windows, Version 21.0 (Armonk, NY: IBM Corp.) The MFPDI total score and sub-domains were reported as means, accompanied by 95% two-sided confidence intervals. Tests for continuous variables were carried out as appropriate to their distributions (t-test for parametric and Mann-Whitney for non-parametric data). Chi-squared tests were carried out between groups for the survey responses at the 5% significance level. Data were analysed using logistic regression with odds of being in work as opposed to reporting work non-participation being the dichotomous outcome variable, with both univariable and multivariable associations modelled.

## Results

One hundred and eighty-two people with SLE completed the questionnaire. Seventy-one of those 182 (39%) respondents were excluded as they were in neither a paid/ voluntary job nor long-term sick/retired due to SLE or foot problems. Statistical comparison of this group (*n* = 71) with the remaining 111 respondents for age in categories (in work most frequent age category = 4 (older); not in work most frequent age category 3; *p* < 0.001), duration of disease (in work mean duration 13 ± 9 (1–36) years; not in work mean duration 16 ± 10 (18–81) years; *p* = 0.596), and BMI (in work mean BMI 27 ± 5 (16–44); not in work mean BMI 28 ± 8 (18–42); *p* = 0.679), suggested that clinically the excluded participants were not significantly different but that they were older. This was not unexpected, given that these respondents are most likely retired due to age, not for a health reason.

Having excluded the 71, the remaining 111 respondents were eligible for further analyses of working status. Seventy-nine (71%) respondents reported that they were currently in paid or voluntary employment as compared with 32 (29%) respondents who reported work non-participation due to SLE and foot symptoms.

Comparison between those currently working and those currently reporting work non-participation showed no differences for ethnicity (*p* = 0.515) or BMI (*p* = 0.817) in univariate analyses, as shown in Table 1. Those who reported work non-participation were more likely to have a longer disease duration (*p* = 0.008) and there was a statistically significant effect of age between the groups (*p* = 0.048). When adjusted for age using multivariable logistic regression, duration of disease no longer remained a statistically significant factor (*p* = 0.132).

**Table 1:** A comparison of demographic characteristics between those in work and those reporting work non-participation

Respondent characteristic	Number of respondents in work with symptom present (n) (max group size = 79)	Number of respondents not in work with symptom present (n) (max group size = 32)	Statistical difference between groups
Age range			0.001
18–29	8	3	
30–39	15	1	
40–49	29	8	
50–59	24	10	
60–69	2	7	
70+	0	2	
Ethnicity			0.515
White	63	22	
Black	3	3	
Asian	6	2	
Other	4	3	
BMI			0.817
Underweight	1	0	
Optimal	35	11	
Overweight	16	5	
Obese	20	10	
Current smoker	11	4	1.000

A comparison of people currently working with those reporting work non-participation is shown in Table 2. All foot symptoms were more frequently reported amongst those reporting work non-participation than those currently employed. After adjustment for age, those not in work were statistically significantly more likely to report previous foot ulceration (OR 3.47, 95% CI: 1.24–9.76, *p* = 0.018) difficulty walking (OR 3.15, 95% CI: 1.16–8.56, *p* = 0.024) and swelling of the foot/ankle (OR 2.91, 95% CI: 1.03–8.13, *p* = 0.041).

**Table 2:** A comparison of the prevalence of foot symptoms and MFPDI scores in relation to work

		In work *N* = 79		Reporting work non-participation *N* = 32		OR (95% CI)	*P*-value
		N Reported	% of employed	N Reported	% of LTS/ Retired		
Raynaud’s in past		42	*53.8%*	19	*59.4%*	1.25 (0.54–2.89)	0.265
Calf pain in past		44	*55.7%*	22	*68.8%*	1.75 (0.73–4.17)	0.107
Calf night pain in past		59	*74.7%*	27	*87.1%*	2.29 (0.71–7.34)	0.065
Loss of feeling in past		10	*12.8%*	11	*34.4%*	3.56 (1.33–9.55	0.053
Ulcer in past		16	*20.5%*	14	*43.8%*	3.01 (1.24–7.33)	0.018
Hard skin in past		56	*72.7%*	27	*84.4%*	2.03 (0.69–5.99)	0.165
Ingrown toenail in past		30	*38.0%*	16	*51.6%*	1.74 (0.75–4.03)	0.264
Reported Rash/Blisters		26	*32.9%*	13	*41.9%*	1.47 (0.63–3.46)	0.699
Difficulty walking in past		25	*31.6%*	18	*58.1%*	2.99 (1.27–7.04)	0.024
Swelling in past		37	*46.8%*	22	*71.0%*	2.78 (1.14–6.78)	0.041
Stiffness in past		62	*79.5%*	28	*93.3%*	3.61 (0.78–16.79)	0.063
Joint pain in past		64	*82.1%*	28	*93.3%*	3.06 (0.65–14.38)	0.155
Change in Foot shape in past		26	*33.8%*	12	*42.9%*	1.47 (0.61–3.57)	0.710
MFPDI function score MAX 20	Mean	6.44		12.10			< 0.001
MFPDI pain score MAX 10	Mean	3.05		4.52			0.018
MFPDI foot appearance score MAX 4	Mean	0.72		1.57			0.038
MFPDI Total MAX 34	Mean	10.16		18.13			< 0.001

Results shown in table two include those adjusted for age

Overall, people those reporting work non-participation had higher mean MFPDI scores (18.13, 95% CI: 14.85–21.41) than those currently employed (10.16, 95% CI: 8.11–12.21) indicating more severe foot problems. Moreover, the same was found for each of the three MFPDI constructs (1. mobility, 2. pain, 3. personal appearance). After adjustment for age using a multivariate logistic regression model (Table 2) the MFPDI scores remained statistically significantly different across all constructs.

## Discussion

This study found that 29% of people with SLE eligible to work report work non-participation because of SLE and lower limb or foot problems. Moreover, comparison to those who are currently working with those who are not shows a greater number of a range of foot problems and significantly higher MFPDI scores. The only demographic factor associated with work non-participation within this dataset was age. However, work non-participation was associated with: ever having foot ulceration; swelling of the foot/ankle; and poor mobility/difficulty walking, even after adjustment for age.

Our finding that work non-participation affects at least 29% of those eligible to work is consistent with the results from other studies of people with SLE [4, 12–14]. Demographic risk factors for work non-participation that have been identified by other researchers include age, ethnicity, lower educational attainment, lower social class, and disease-specific risk factors include disease duration, activity, pain, fatigue, depression, cognitive function, neuropsychiatric manifestations and damage [15]. To our knowledge however, no other study has explored the impact of foot symptoms on work participation and in particular, it would appear that poor mobility may have an important effect on work participation. As with other studies, our data are cross-sectional so that we are unable to draw conclusions about causation. However, it is of interest that high MFPDI scores and foot ulceration or swelling of the foot/ankle were significantly associated with risk of work non-participation and we suggest that future research about work participation in people with SLE should consider poor mobility and foot symptoms as well as other factors in prospective studies.

It is of interest that the MFPDI scores amongst those participating in work still suggest that foot symptoms are prevalent even when people are sustaining their work. This of course has important implications clinically, particularly if it can be demonstrated that baseline scores predict future work non-participation, which may allow for targeted personalised care planning. It suggests that foot health should receive greater emphasis within the clinic and that rheumatology clinicians should regularly ask about foot symptoms and examine the foot or involve podiatrists in the support of people with SLE.

It should be borne in mind that there were a number of limitations to this study. In particular, the numbers of participants eligible to work who reported work non-participation was relatively small and the study may have consequently been relatively under-powered. In light of that, it is in fact striking that statistically significant associations were identified for those reporting work non-participation. Moreover, because of the relative rarity of SLE as a condition, this is a large cohort when compared to the available literature on SLE and work participation. Another difficulty inherent in the design of this study was that no information is available as to the number of non-responders or their clinical characteristics or work participation. Consequently, we are unable to conjecture about the size or nature of any responder bias. It is possible that people with SLE who perceived themselves as having particular problems with their foot health were more motivated to participate than those unaffected by such symptoms. This study was designed to collect a minimum of personal information to reduce the burden upon respondents; however this does mean that there is limited information regarding disease severity or clinical manifestations that do not affect the feet. In contrast, a particular strength of this study is the clarity over the case definition of work participation that has been used. Baker and Pope [4] found this to be a very heterogeneous literature with wide variation in the types of case definition that had been used and it is important that research going forwards is consistent in choosing measureable and reliable measures of work participation.

## Conclusion

To our knowledge this is the first study to examine the impact of foot symptoms on work amongst people with SLE. Twenty-nine% of people with SLE eligible to work reported work non-participation because of SLE and lower limb or foot problems. Our results suggest that foot health and poor mobility may be important contributors to a persons’ ability to remain in work although further prospective research is recommended. This work has important implications clinically, particularly if it can be demonstrated that baseline scores predict future work non-participation, which may allow for targeted personalised care planning. It suggests that foot health should receive greater emphasis and that clinicians should examine and discuss the impact of poor foot health or involve podiatrists in the support of people with SLE.

## Abbreviations

MFPDI: Manchester Foot Pain and Disability Index
MSK: Musculoskeletal
SLE: Systemic Lupus Erythematosus
UK: United Kingdom
